# The ecological role of bacterial seed endophytes associated with wild cabbage in the United Kingdom

**DOI:** 10.1002/mbo3.954

**Published:** 2019-11-13

**Authors:** Olaf Tyc, Rocky Putra, Rieta Gols, Jeffrey A. Harvey, Paolina Garbeva

**Affiliations:** ^1^ Department of Microbial Ecology Netherlands Institute of Ecology (NIOO‐KNAW) Wageningen The Netherlands; ^2^ Department of Internal Medicine I Goethe University University Hospital Frankfurt Frankfurt Germany; ^3^ Laboratory of Entomology Wageningen University Wageningen The Netherlands; ^4^ Hawkesbury Institute for the Environment Western Sydney University Penrith Australia; ^5^ Department of Terrestrial Ecology Netherlands Institute of Ecology Wageningen The Netherlands; ^6^ Department of Ecological Sciences Section Animal Ecology VU University Amsterdam Amsterdam The Netherlands

**Keywords:** bacteria, endophytes, fungal pathogens, insect herbivory, plant growth, plant resistance, plant‐insect interactions, seed germination

## Abstract

Endophytic bacteria are known for their ability in promoting plant growth and defense against biotic and abiotic stress. However, very little is known about the microbial endophytes living in the spermosphere. Here, we isolated bacteria from the seeds of five different populations of wild cabbage (*Brassica oleracea* L) that grow within 15 km of each other along the Dorset coast in the UK. The seeds of each plant population contained a unique microbiome. Sequencing of the 16S rRNA genes revealed that these bacteria belong to three different phyla (Actinobacteria, Firmicutes, and Proteobacteria). Isolated endophytic bacteria were grown in monocultures or mixtures and the effects of bacterial volatile organic compounds (VOCs) on the growth and development on *B. oleracea* and on resistance against a insect herbivore was evaluated. Our results reveal that the VOCs emitted by the endophytic bacteria had a profound effect on plant development but only a minor effect on resistance against an herbivore of *B. oleracea*. Plants exposed to bacterial VOCs showed faster seed germination and seedling development. Furthermore, seed endophytic bacteria exhibited activity via volatiles against the plant pathogen *F. culmorum*. Hence, our results illustrate the ecological importance of the bacterial seed microbiome for host plant health and development.

## Graphical abstract

Here, we isolated bacteria from the seeds of five different populations of wild cabbage (*Brassica oleracea* L.) that grow within 15 km of each other along the Dorset coast in the UK. The seeds of each plant population contained a unique set of bacteria. We tested the effect of these bacterial endophytes on the growth and development on wild cabbage and on resistance against an insect herbivore (*Mamestra brassicae*) and a plant pathogenic fungus (*Fusarium culmorum*). Our results reveal the endophytic bacteria had a profound effect on plant development and health but only a minor effect on resistance against an herbivore insect.

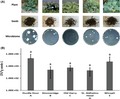

## 1 INTRODUCTION

Plants are involved in intimate interactions with microbes throughout their entire life cycle, and these interactions are essential for the growth and health of the plants. Endophytes are nonpathogenic microorganisms that inhabit plants without causing them any harm (Hardoim, Hardoim, Overbeek, & Elsas, 2012; Rosenblueth & Martinez‐Romero, 2006). Endophytic microorganisms live in an intimate relationship with their host throughout many generations (Johnston‐Monje & Raizada, 2011). Many endophytic bacteria are known for their growth‐promoting effect on plants and for priming plant immunity by triggering induced systemic resistance (ISR) and/or induced systemic tolerance (IST) (Hardoim, Overbeek, & Elsas JDv., 2008; Nass et al., 2005; Ryu et al., 2004, 2003). Several studies have shown that endophytes can enhance antagonistic activities of plant pathogens and aid plants against biotic and abiotic stresses (Berg et al., 2005; Cosme et al., 2016; Egamberdieva, Davranov, Wirth, Hashem, & Abd_Allah EF., 2017; Grover, Ali, Sandhya, Rasul, & Venkateswarlu, 2011 *)*. Seed endophytes are known to be vertically transmitted from mother plants to their offspring (Hardoim, Overbeek and Elsas, 2008; Frank, Saldierna Guzmán and Shay, 2017; Nelson, 2018), suggesting that the role of seed endophytes is highly crucial, especially at the early stage of host plant development (Berg & Raaijmakers, 2018; Nelson, 2018; Truyens, Weyens, Cuypers, & Vangronsveld, 2015). However, little is known so far about the ecological role of the seed endophytes and seed microbiome.

Essential for plant–microbe interactions and communication are secondary (or specialized) metabolites produced by either partners. Both plants and microorganisms produce a wide variety of secondary metabolites including volatile and nonvolatile compounds. Only in the past few decades, the functional role of microbial volatiles has been increasingly acknowledged and investigated (Sharifi & Ryu, 2018). Plant‐associated microorganisms produce an vast array of volatiles ranging from inorganic compounds, such as CO_2_, NH_3,_ and HCN to a plethora of organic compounds, such as terpenes, ketones, alcohols, alkenes, alkanes, esters, and sulfur‐derived compounds (Kanchiswamy, Malnoy, & Maffei, 2015; Schulz & Dickschat, 2007). Volatile organic compounds (VOCs) are compounds with a small molecular weight (<300 da). They can easily evaporate and travel through air and water‐filled pores in the soil (Penuelas et al., 2014; Schulz & Dickschat, 2007). So far, the most well studied VOCs emitted by soil microorganisms are terpenes, nitrogen‐based compounds like indole and sulfur‐containing compounds like dimethyl disulfide (Tyc, Song, Dickschat, Vos, & Garbeva, 2017). Soil microorganisms can employ volatiles as info chemicals, growth stimulants, growth inhibitors, and inhibitors of quorum sensing (Chernin et al., 2011; Effmert, Kalderas, Warnke, & Piechulla, 2012; Kai et al., 2009; Kim, Lee, & Ryu, 2013). Moreover, interspecific interactions of phylogenetically different bacteria can also alter the volatile blend composition, affecting the activity of volatiles (Garbeva, Hordijk, Gerards, & Boer, 2014; Tyc et al., 2015). The effects of the emitted microbial VOCs on the host plants and their antagonists can vary from negative, positive to neutral (van Dam, Weinhold, & Garbeva, 2016). For instance, plant‐growth promoting effects were reported for volatiles emitted by bacteria (Park, Dutta, Ann, Raaijmakers, & Park, 2015; Ryu et al., 2003) and fungi (Cordovez et al., 2017). In addition, volatiles from an endophyte of maize (*Zea mays*), *Enterobacter aerogenes* have been shown to alter the host plant's resistance to a fungal pathogen and an insect pest (D'Alessandro et al., 2014), suggesting that volatiles also exhibit plant protection against a broad range of attackers. Interestingly, volatiles emitted by the nectar‐inhabiting yeast *Metschnikowia reukaufii* influenced the nectar preference of a generalist bee (Rering, Beck, Hall, McCartney, & Vannette, 2018). However, it is unknown so far whether volatiles emitted by seed endophytes in particular benefit the associated host plant and whether interspecific interactions between endophytes change volatile emission with consequences for the host in terms of growth, development, and resistance.

Here, we aimed to investigate the potential role of volatiles produced by seed endophytic bacteria associated with wild cabbage (*Brassica oleracea* L.) on plant growth, development and resistance against a leaf chewing insect herbivore and two pathogenic fungi. These wild cabbage populations are considered to be the ancestors of current cultivated cabbage. Seeds originated from five populations growing along the rugged coastline of Dorset, United Kingdom (Gols, Dam, Raaijmakers, Dicke, & Harvey, 2009; Van Geem, Harvey, Cortesero, Raaijmakers, & Gols, 2015; Wichmann, Alexander, Hails, & Bullock, 2008). Previous work has shown that there is considerable population‐related variation in the expression of primary and secondary metabolites (glucosinolates) in British populations of wild cabbage. These differences have an effect on the behavior and development of several species of insect herbivores and their natural enemies associated with these plants both in the laboratory and in the field (Gols, Bullock, Dicke, Bukovinszky, & Harvey, 2011; Gols et al., 2008; Harvey, Dam, Raaijmakers, Bullock, & Gols, 2011; Moyes, Collin, Britton, & Raybould, 2000; Newton, Bullock, & Hodgson, 2009; Van Geem et al., 2015). However, this previous research ignored the possibly important role played by the plant microbiome on plant traits that affect growth, fitness, and defense. We hypothesize that seeds of wild cabbage contain cultivable endophytic bacteria whose volatiles are beneficial for the host plant. Here, we aim to isolate endophytic bacteria from five different populations of wild cabbage plant populations. We hypothesize that the five different plant populations harbor different endophytic bacterial strains, each producing its specific volatile blend, which in turn differentially affect their interaction with the host plant.

## 2 MATERIALS AND METHODS

### 2.1 Seeds and extraction of endophytic bacteria

**Figure 1:**
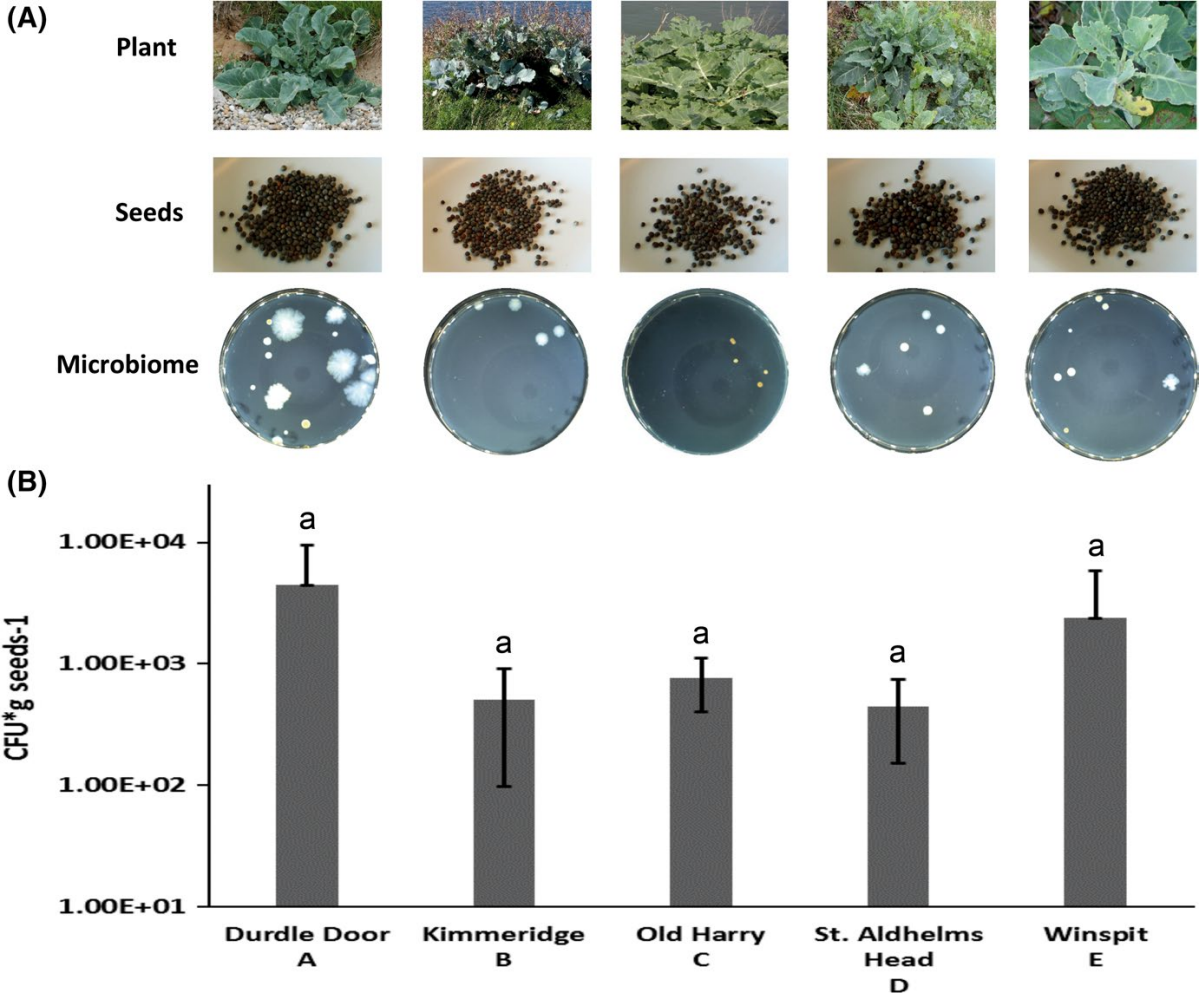
The five different wild cabbage plant (*B. oleracea*) populations grown at their natural location, their seeds, and their isolated cultivable microbiome. (A) Overview of the five different used plant populations, seeds, and the isolated microbiome from 1 gram of seeds. (B) Number of bacterial colony‐forming units (CFU) obtained from 1 gram of surface‐sterilized seeds of each plant population. Bars represent standard deviation (*SD*). No significant differences in CFU/g seed were observed among the seeds of the five plant populations (ONE‐WAY ANOVA post hoc Tukey tests). The same letter above the bars indicates no significant difference between the samples with *p* > .05

**Figure A1:**
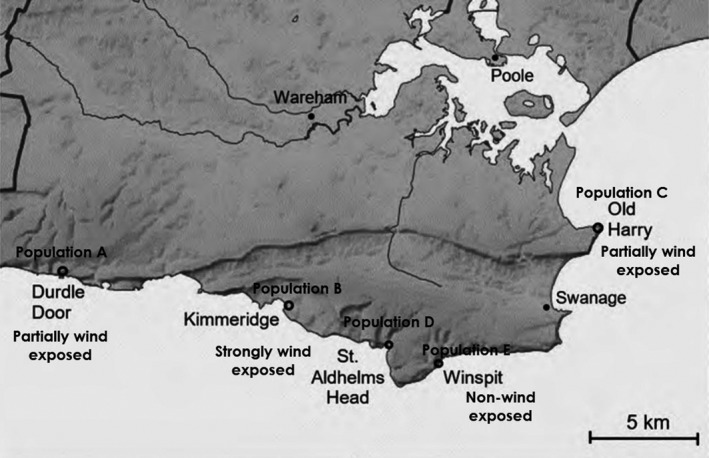
Origin of the five populations of wild cabbage *B. oleracea* that grow within a 15‐km distance along the Dorset coast in the United Kingdom (Figure adapted by Van Geem *et al.,* 2015) [Correction added on 4 December 2019 after first online publication: Figure A1 has been moved from Supporting Information to the Appendix section]

Seeds of five different populations of wild cabbage *Brassica oleracea* collected from the Dorset coast in the UK were used in this study: A: Durdle Door (DD; 50˚62’N, 2˚27’W), B: Kimmeridge (KIM; 50˚35’N, 2˚03’W), C: Old Harry (OH; 50˚38’N, 1˚55’W), D: St. Aldhelms Head (SAH; 50˚69’N, 2˚05’W), and E: Winspit (WIN; 50˚34’N, 2˚02’W) (Van Geem et al., 2015) (Figure 1a; Figure A1). Seeds were surface‐sterilized by a modified protocol by Araujo et al. (2002). To this end, seeds (1 g) of each plant population were subsequently incubated for 3 min in 2% NaOCl, 3 min in 80% ethanol, and rinsed five times with sterile distilled water. The sterilized seeds were transferred to a sterile mortar with 1 ml of 10 mM phosphate buffer (pH 6.5) and crushed using a sterile pestle. A volume of 100 µl was taken and transferred to 900 µl of 10 mM phosphate buffer. A serial dilution was made from this solution, and each dilution was plated in triplicates on 1/10th TSBA plates (5.0 g/L NaCl, 1.0 g/L KH_2_PO_4_; 3 g/L Oxoid Tryptic Soy Broth; and 20 g/L BACTO agar, pH 6.5) (Tyc et al., 2015). Plates were incubated for one week at 24°C and examined regularly for visible bacterial growth.

### 2.2 Enumeration of bacterial colony‐forming units (CFU) and preparation of glycerol stocks

For the enumeration of colony‐forming units (CFU) of the isolated endophytic bacteria an aCOlyte Colony Counter (Don Whitley Scientific, Meintrup DWS Laborgeräte GmbH, Germany) was used. After one week of incubation, the CFUs of each petri dish containing the bacteria were enumerated. The CFU numbers were based on three replicates per dilution series per plant population. Single bacterial colonies were picked from plates and transferred to 10 ml liquid 1/10th Tryptic Soy Broth (TSB) (5.0 g/L NaCl, 1.0 g/L KH_2_PO_4_; and 3 g/L Oxoid Tryptic Soy Broth) and incubated overnight at 24°C, 190 rpm. The next day a volume of 750 µl culture was mixed with 750 µl 50% (v/v) glycerol. Prepared glycerol stocks were transferred to a −80°C freezer for long time storage.

### 2.3 Taxonomic identification of endophytic bacteria by 16S rRNA PCR

For molecular identification of the isolated endophytic bacteria, colony PCRs were performed. For this, a single colony of each bacterial isolate was collected from plate with a disposable inoculation loop (VWR international B.V., Amsterdam, the Netherlands Cat# 50806–404) and transferred to a GoTaq® 50 µl PCR‐ master mix reaction (Promega Corp. Madison, USA cat# M7122). For 16S rRNA gene amplification, forward primer 27F (5’‐ AGA GTTT GAT CMT GGC TCAG −3’), reverse primer 1492R amplifying ~ 1,465 bp from the 16S rRNA gene were used (Edwards, Rogall, Blocker, Emde, & Bottger, 1989; Lane, 1991) (modified). All PCR reactions were performed on a Bio‐Rad C1000 Touch Thermocycler (Bio‐Rad Laboratories, Veenendaal, the Netherlands) with the following settings: initial cycle 95°C for 2.5 min, 30 cycles of 94°C for 30 s, 55°C for 45 s, 72°C for 1 min, and a final round of amplification at 72°C for 5 min. After amplification, a volume of 5 µl of each PCR reaction was loaded on a 1% (w/v) agarose gel and checked after electrophoresis for presence of correct‐sized PCR fragments. Positive PCR products were cleaned using the Qiagen PCR purification kit (Cat# 28,104; Qiagen Benelux BV, Venlo, the Netherlands) and sent to Macrogen (Macrogen Europe, Amsterdam, the Netherlands) for sequencing. The obtained sequences of the 16S rRNA gene were examined for quality and trimmed to approximately the same size (~700 bp) using BioEdit 7.2.5 (Hall, 1999). For taxonomic identification, the 16S rRNA gene sequences were aligned and compared against those available in the NCBI database by using BLASTN algorithm (blast.ncbi.nlm.nih.gov) (Altschul et al., 1997). The trimmed sequences were aligned using ClustalW algorithm and exported as FASTA sequence and phylip files. A Maximum‐likelihood phylogenetic tree was created based on the alignment of the partial 16S DNA sequence based on the alignment of approximately 700 bp of the 5′ 16S rRNA gene of each isolate. Outgroup: *Anabaena circinalis*. 33–8. The Alignments and the tree were generated with ClustalW and bootstrap analysis was performed with 10,000 resamplings. Phylogenetic tree images were created by using the phylogeny.fr platform (http://www.phylogeny.fr) (Dereeper et al., 2008) using standard settings. The sequences obtained during this study are submitted to NCBI GenBank under submission number SUB5675460 and the accession numbers MN079062 – MN079072 (Table 1).

**Table 1:** Organisms used in this study

Organism	Population	Phylum/Order	Genbank	BLAST similarity %	Reference
**Endophytic bacteria**					
*Micrococcus aloeverae* isolate A1	*Durdle Door*	Actinobacteria	MN079062	95%	This publication
*Micrococcus yunnanensis* isolate A3	*Durdle Door*	Actinobacteria	MN079063	96%	This publication
*Aeromicrobium fastidiosum* isolate A4	*Durdle Door*	Actinobacteria	MN079064	97%	This publication
*Pseudomonas marginalis* isolate B1	*Kimmeridge*	Proteobacteria	MN079065	97%	This publication
*Gordonia bronchialis* isolate C4	*Old Harry*	Actinobacteria	MN079067	98%	This publication
*Pseudomonas azotoformans* isolate D1	*St. Aldhelms Head*	Proteobacteria	MN079066	99%	This publication
*Stenotrophomonas rhizophila* isolate D5	*St. Aldhelms Head*	Proteobacteria	MN079068	96%	This publication
*Pseudomonas orientalis* isolate E8	*Winspit*	Proteobacteria	MN079069	98%	This publication
*Chryseobacterium indoltheticum* isolate E9	*Winspit*	Bacteroidetes	MN079070	96%	This publication
*Pantoea agglomerans* isolate E44	*Winspit*	Proteobacteria	MN079071	98%	This publication
*Stenotrophomonas chelatiphaga* isolate E50	*Winspit*	Proteobacteria	MN079072	93%	This publication
**Fungal organism**					
*Rhizoctonia solani* AG2.2IIIB	–	Basidiomycota	KT124637	—	Garbeva et al. (2011)
*Fusarium culmorum* PV	–	Ascomycota	–	—	Garbeva, Hordijk, Gerards, and Boer (2014)
**Insect organism**					
*Mammestra brassicae*	–	Arthropoda	–		Gols et al. (2008)
**Plant organism**					
*Brassica oleracea*	–	Brassicales	–		Gols et al. (2008)

### 2.4 Effects of bacterial volatiles on seed germination and early plant development

To assess the effect of volatiles emitted by endophytic bacterial on plant growth and development, seeds of each of the five plant populations (coded A‐E, Figure 1) were grown in two different experiments in the presence or absence of volatiles emitted by the following bacterial isolates: *Pseudomonas marginalis* B1, *Pseudomonas orientalis* E8, *Pseudomonas azotoformans* D1, *Stenotrophomonas rhizophila* D5, and *Pantoea agglomerans* E44 (the letters B to E refer to the plant population from which the bacteria were isolated (Figure 1). The seeds were surface‐sterilized as described above, dried on filter papers in a flow cabinet for 15 min, and stratified for 3 days at 4°C. An overnight inoculum of each bacterial isolate (Table 1) was prepared. For this, a single colony of each bacterial isolate was picked from plate and grown in 20 ml 1/10th Tryptic Soy Broth at 190 rpm and 20°C. Each bacterial inoculum was diluted to an OD_600_ of 0.005 (monoculture or mixtures) in 20 ml 10 mM phosphate buffer. Fifty µl was plated on 1/10th Tryptic Soy Agar (pH = 6.5) in a two‐compartment Petri dish (9 cm diameter; Greiner bio‐one B.V., Alphen a/d Rijn, the Netherlands, Cat# 635,102) and incubated at 20°C for 48 hr. After three days of stratification, 8 seeds of each plant population were placed on 0.8% plant agar medium (P1001 Duchefa Biochemie, pH = 5.8) opposite the inoculated bacterial isolates in the two‐compartment Petri dish. Plates containing the bacteria and seeds were incubated for a week. For the control, seeds were placed on one side of the two‐compartment Petri dish without bacterial inoculum being added to the growth medium. All Petri dishes were sealed with Parafilm and stored in climatic chamber (20°C; 180 µ mol light/m^2^/s at plant level; 16:8 hr (light: dark); 60%–70% R.H.). Images were captured starting from the 3rd day to the 7th day to record radicle emergence, primary root length. For the estimation of the seedling fresh weight on days three, five and seven the seedlings were weighed on a microbalance (Mettler‐Toledo MT5 Electrobalance). Primary root length of seedlings (cm) was analyzed using *SmartRoot* plugin in *Fiji*, image analysis software (Schindelin et al., 2012). Three technical replicates were prepared.

### 2.5 Effects of bacterial volatiles of P. marginalis, P. azotoformans, and the mixture of both bacteria on plant growth

Seeds of the Winspit (E) plant population were used to assess the effect of bacterial volatiles emission on *B. oleracea* growth and plant dry mass after four weeks. Seeds of this plant population were used as this plant population showed in earlier experiments significant defense response to insect herbivores (Gols et al., 2008). The plants were grown in presence or absence of volatiles emitted by *P. marginalis*, *P. azotoformans,* and the mixture of both bacteria, which were the most abundant culturable bacteria in the seeds of the five tested *B. oleracea* populations. Bacterial suspensions were prepared and seeds were treated as described above. In total, 16 Petri dishes (4 per treatment (3) and the control) were prepared and incubated for one week. For the control, seeds were placed on one side of the two‐compartment Petri dish without added bacterial inoculum (*n* = 4). All Petri dishes were sealed with Parafilm and stored in climatic chamber (20°C; 300 µ mol light m^‐2^s^‐1^ at plant level; 16:8 hr (light:dark)) for seed germination and pregrowth of the plants. A total of 64 one‐week‐old seedlings that were either exposed or not exposed to bacterial volatiles were transferred to 15‐ml tubes containing 0.8% plant agar medium P1001 Duchefa Biochemie (pH = 5.8), at half‐strength Murashige & Skoog (MS) medium (2.165 g/L) (Murashige & Skoog, 1962) including vitamins supplemented with 0.5% sucrose. The tubes containing the seedlings were transferred to a BioAssay tray (Nunc™ Square BioAssay Dishes Cat# 240,845, ThermoFisher Scientific, L = 245 mm; W = 245 mm; H = 25 mm) and sealed with a gas permeable adhesive plaster (Kruidvat sparadrap sensitive, Kruidvat, the Netherlands). Per treatment, four bioassay trays were used and four plants were placed per bioassay tray. The bioassay trays were transferred to climate‐controlled growth chamber (20°C; 300 µ mol light/m^2^/s at plant level; 16:8 hr (light: dark)). After a total incubation time of four weeks, the plants were harvested. After determination of their fresh weight (Sartorius BA‐160P microbalance), shoots and roots were separated per plant, dried in an oven at 60°C for four days and reweighed.

### 2.6 Effects of bacterial volatiles on fungal growth (mycelial expansion)

**Figure A2:**
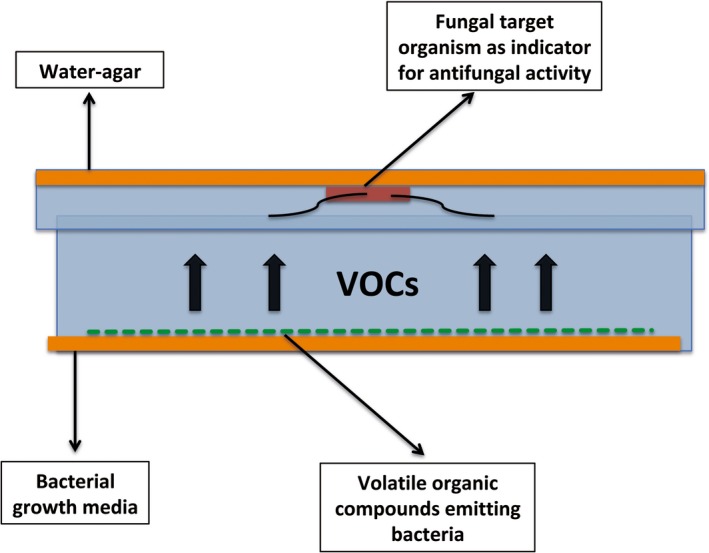
Used top‐bottom‐Petri dish system for experiments to elucidate fungal inhibitory capacities of the produced bacterial volatiles [Correction added on 4 December 2019 after first online publication: Figure A2 has been moved from Supporting Information to the Appendix section]

To test the effect of the emitted bacterial volatiles on fungal hyphal extension, the two plant pathogenic model fungi, *Rhizoctonia solani* (AGII) 2.2IIIB (Garbeva, Silby, Raaijmakers, Levy, & Boer, 2011) and *Fusarium culmorum* were used (de Rooij‐van der Goes, 1995)*.* The fungi were precultured on 1/5th Potato Dextrose Agar (PDA) (29 g/L Oxoid CM 139) (Fiddaman & Rossall, 1993) and incubated at 24°C for 7 days prior to the experiment. The assays were performed in Petri dishes (9 cm diameter, Greiner bio‐one B.V., Alphen a/d Rijn, the Netherlands, Cat# 633,180), containing a top and a bottom growth area (Figure A2). For the assay a single colony of either *Pseudomonas marginalis* B1, *Pseudomonas orientalis* E8, *Pseudomonas azotoformans* D1, *Stenotrophomonas rhizophila* D5, or *Pantoea agglomerans* E44 was picked and grown overnight in 20 ml 1/10th TSB media. For the inoculation of the bottom of the Petri dish, 100 µl of bacterial suspensions (OD 0.005) in 10 mM phosphate buffer (pH 6.5) containing ~ 10^5 cells/mL were spread on 20 ml 1/10th tryptic soy broth agar (TSBA). In the lid of the Petri dish, 12.5 ml of water‐agar medium (WA) (20 gL^‐1^ BACTO agar) was added and inoculated in the middle with a 6‐mm‐diameter PDA agar plug containing *R.solani or F.culmorum* hyphae. The plates were sealed with Parafilm and incubated at 24°C for five days. This allowed us to test fungal exposure to the volatiles produced by the bacteria grown in the bottom compartment without the fungi being in direct physical contact with the bacteria. On the fifth day, the extension of the hyphae was measured and compared to the hyphae extension in the control plates (fungi exposed to 1/10th TSBA growth medium without bacteria). For the analysis, digital photographs were taken. The digital images were analyzed using the AXIO VISION v4.8 imaging Software (Carl Zeiss Imaging Solutions GmbH).

### 2.7 Effects of bacterial volatile exposure on plant herbivory resistance

We also tested the effect of volatiles produced by *P. marginalis*, *P. azotoformans,* and the mixture of both bacteria on plant resistance against a chewing insect herbivore, *Mamestra brassicae*. The bacteria were grown in mono or mixed cultures. Bacterial cultures and seeds were prepared and added to the two‐compartment Petri dish as described above. After 3 days of stratification, 8 seeds from the Winspit (E) population were placed on the other side of the Petri dish containing 0.8% plant agar medium P1001 Duchefa Biochemie (pH = 5.8). For the control, seeds were placed on one side of the two‐compartment Petri dish without adding the bacterial inoculum. All Petri dishes were sealed with a gas permeable adhesive plaster (Kruidvat sparadrap sensitive, Kruidvat, the Netherlands) and stored in a climate chamber for 5 days for seed germination and pregrowth of the plants (20°C; 180 µ mol light/m^2^/s at plant level; 16:8 hr (light: dark); 60%–70% R.H.). Five‐day‐old seedlings were transferred to 15‐ml tubes containing 0.8% plant agar medium P1001 Duchefa Biochemie (pH = 5.8), at half‐strength Murashige & Skoog (MS) medium (2.165 g/L) (Murashige & Skoog, 1962) including vitamins supplemented with 0.5% sucrose. The tubes were incubated and continuously exposed to bacterial volatiles in a Bioassay tray (Nunc™ Square Bioassay Dishes Cat# 240,845, ThermoFisher Scientific, L = 245 mm; W = 245 mm; H = 25 mm) that were placed in climate‐controlled growth chamber for 24 days before the larvae were introduced. Following incubation, the plants were infested with *M. brassicae* neonates L1 (5 larvae per plant) and incubated in an insect growth chamber (20°C; 180 µ mol light/m^2^/s at plant level; 16:8 hr (light: dark)) for 7 days. Eggs of *M. brassicae* were obtained from the Laboratory of Entomology (Wageningen University, the Netherlands. The Wageningen culture has been reared for many generations on *Brassica oleracea,* cultivar Cyrus, in a controlled growth chamber (22 ± 2°C; 16:8 hr (light: dark); 40%–50% R.H.). Larval fresh biomass was measured on a microbalance (Mettler‐Toledo MT5 Electrobalance) at two time points (day 3 and 7) as a proxy for plant resistance. In addition, larval survival was assessed by counting the number of live larvae on each plant at the same two time points.

### 2.8 Trapping, analyzing, and identifying of bacterial volatile organic compounds

For trapping of the volatile organic compounds emitted by the endophytic bacteria a volume of 100 µl inoculation suspension (OD_600_ of 0.005) of each bacterial isolate was spread on 1/10th Tryptic Soy Broth Agar (TSBA) (20 ml) in special glass Petri dishes designed for headspace volatile trapping (P Garbeva et al., 2014). The Petri dishes were closed by a lid with an outlet connected to a steel trap containing 150 mg Tenax TA and 150 mg Carbopack B (Markes International Ltd., Llantrisant, UK). All treatments were inoculated in triplicate. The volatiles were collected after 72 hr of incubation by adding the Tenax steel traps to the outlet of the glass petri dish overnight. The Tenax traps were afterward stored at 4°C until GC‐Q‐TOF analysis. Volatile organic compounds were desorbed from the traps using a thermo desorption unit (Unity TD‐100; Markes International Ltd., Llantrisant, UK) at 210°C for 12 min (He flow 50 ml/min) and trapped on a cold trap at −10°C. The volatiles were introduced into a GC‐MS‐QTOF (model Agilent 7890B GC and the Agilent 7200A QTOF, Santa Clara, USA) by heating the cold trap for 3 min to 280°C. Split ratio was set to 1:10, and the column used was a 30 × 0.25 mm ID RXI‐5MS, film thickness 0.25 μm (Restek 13424–6850, Bellefonte, PA, USA). The temperature program was as follows: 39°C for 2 min, from 39°C to 95°C at 3.5°C/min, then to 165°C at 6°C/min, to 250°C at 15°C/min, and finally to 300°C at 40°C/min, hold 20 min. The VOCs were detected by the MS operating at 70 eV in EI mode. Mass spectra were acquired in full‐scan mode (30–400AMU, 4 scans/s) and extracted with MassHunter Qualitative Analysis Software V B.06.00 Build 6.0.633.0 (Agilent Technologies, Santa Clara, USA) using the GC‐Q‐TOF qualitative analysis module. The obtained mass spectra were translated to cdf files using Agilent GC AIA Translator VB.07.00 SP2 (Agilent Technolgies, Santa Clara, USA). The created cdf files were imported to MZmine V2.20 (Copyright © 2005–2012 (MZmine Development Team) (Katajamaa, Miettinen, & Oresic, 2006; Pluskal, Castillo, Villar‐Briones, & Oresic, 2010), and compounds were identified via deconvolution (local‐maximum algorithm) in combination with two mass spectral libraries: NIST 2014 V2.20 (National Institute of Standards and Technology, USA http://www.nist.gov) and Wiley 7th edition spectral libraries and by their linear retention indexes (LRI). The LRI values were calculated using an alkane calibration mix before the measurements in combination with AMDIS 2.72 (National Institute of Standards and Technology, USA). The calculated LRI were compared with those found in the NIST and in the in‐house NIOO LRI database. Peak lists containing the mass features of each treatment were exported in csv file format and uploaded to Metaboanalyst V3.5 (http://www.metaboanalyst.ca) (Xia, Sinelnikov, Han, & Wishart, 2015).

## 3 STATISTICAL ANALYSIS

The effect of bacterial volatiles on plant growth and development were statistically analyzed using IBM SPSS Statistics 25. For the analysis of the dry weight ONE‐WAY ANOVA and post hoc TUKEY test were performed. For plant development and seed germination, the explanatory variables in the analyses were exposure treatment, population, and their interaction. For the analysis of radicle emergence (seed germination) a generalized linear model (binomial distribution with a logit link function) was applied. Primary root length and seedling fresh biomass were analyzed using a general linear model followed by a post hoc TUKEY (HSD) test when at least one of the model terms was significant (*p* ≤ .05). To statistically assess the effect of volatiles‐exposed plants on insect performance, data were analyzed separately for each time point (day 3, 5 and 7). Statistical differences on larval biomass were assessed using a general linear model whereas statistical differences on larval survival were analyzed using a generalized linear model (Binary Binomial distribution with a logit link function). Statistical analysis on volatile metabolites data was performed using Metaboanalyst V3.5, http://www.metaboanalyst.ca (Xia et al., 2015). Prior to statistical analysis data normalization was performed via log transformation. To identify significant abundant masses ONE‐WAY‐ANOVA with post hoc TUKEY test was performed between the data sets. To identify important mass features PLSD analysis was performed. Masses were considered to be statistical relevant if FDR values were ≤ 0.05. The effect of bacterial volatiles on fungal growth were statistically analyzed in IBM SPSS Statistics 25 using ONE‐WAY ANOVA and post hoc TUKEY (HSD) test.

## 4 RESULTS

### 4.1 Abundance and phylogenetic analysis of the isolated bacterial endophytes

**Figure 2:**
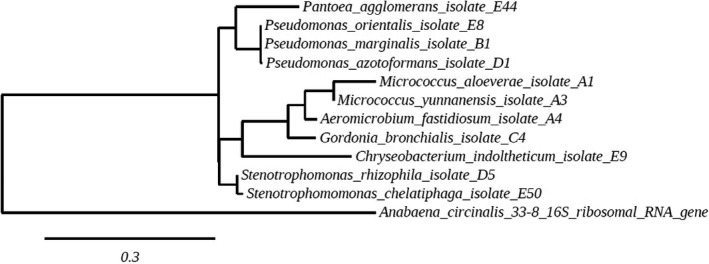
Maximum‐likelihood phylogenetic tree showing the relationship of the isolated endophytic bacteria isolated from the seeds of five populations of B. oleracea. The partial 16S gene tree is based on the assembly of approximately 700 bp of the 5′ 16S rRNA gene. Outgroup: *Anabaena circinalis* 33–8. Alignments and the tree were generated with ClustalW and bootstrap analysis was performed with 10,000 resampling's. Isolates assignation: A: Durdle Door, B: Kimmeridge, C: Old Harry, D: St. Aldhelms Head, E: Winspit

From each plant population, we could isolate different sets of bacteria (Figure 1a). The bacterial colony‐forming units (CFU) we obtained from *B. oleracea* seeds varied per plant population. However, the number of colony‐forming units did not differ statistically significantly (*p* > .05) among the different plant populations. The number of bacterial colony‐forming units (CFU/mL) varied between 4.47 x 10^2^ CFU/g in seeds from St. Aldhelms Head and 4.48 x 10^3^ CFU/g in seeds from Durdle Door. From seeds of the plant population Winspit (plant population E), we were able to obtain an average of 2.38 * 10^3 CFU/g of seed material. From seeds from plant population Old Harry (plant population C), we could obtain an average of 7.58 * 10^2 CFU/g followed by Kimmeridge (plant population B) with 5.05 * 10^2 CFU/g of seeds. The least colony‐forming units per gram of seed material were retrieved from seeds of plant population St. Aldhelms Head (plant population D) with an average of 4.47 * 10^2 CFU/g (Figure1b). In total, 90 bacterial colonies were picked from agar plates and sequenced. The phylogenetic analysis revealed that the bacterial isolates belonged to 11 different species belonging to 3 phyla covering 4 classes Actinobacteria (Actinobacteria), Bacteroidetes (Flavobacteriia), and Proteobacteria (Gamma‐proteobacteria, Alpha‐proteobacteria) Table 1 and Figure 2.

### 4.2 Effects of bacterial volatiles on seed germination, primary root length and plant biomass

**Figure 3:**
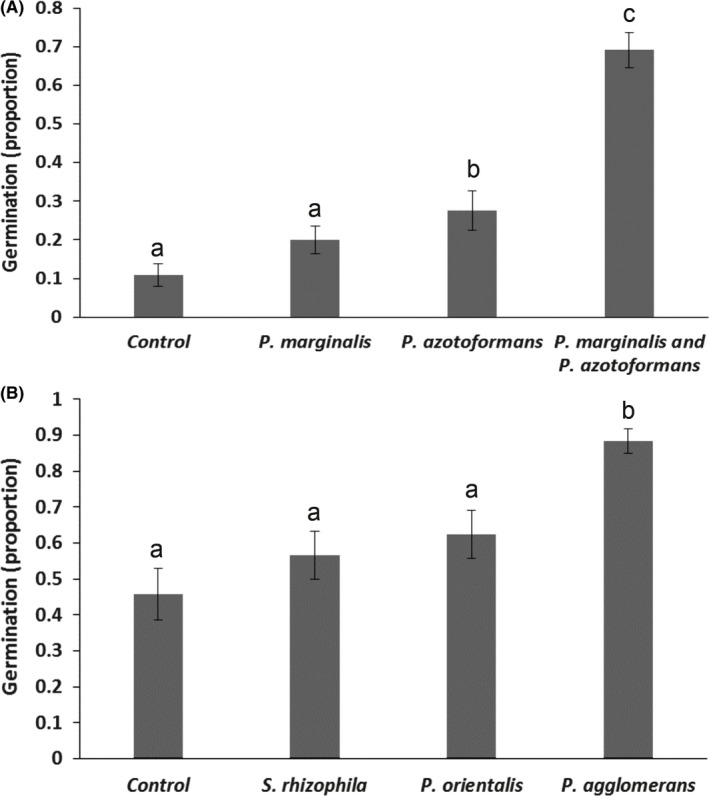
Germination (proportional) of wild cabbage (*B. oleracea*) seeds on the 5th day following continuous exposure to bacterial volatiles emitted by (A) *P. marginalis, P. azotoformans,* and the combination of both compared with the control (*B. oleracea* without exposure to bacterial volatiles). (B) when exposed to bacterial volatiles emitted by monocultures of *S. rhizophila, P. orientalis* and *P. agglomerans* or control (no bacterial volatile exposure) for five days. Significant differences between the treatments and the control are indicated by different letters above bars based on ONE‐WAY ANOVA, post hoc Tukey multiple comparison tests (*n* = 8)

**Figure 4:**
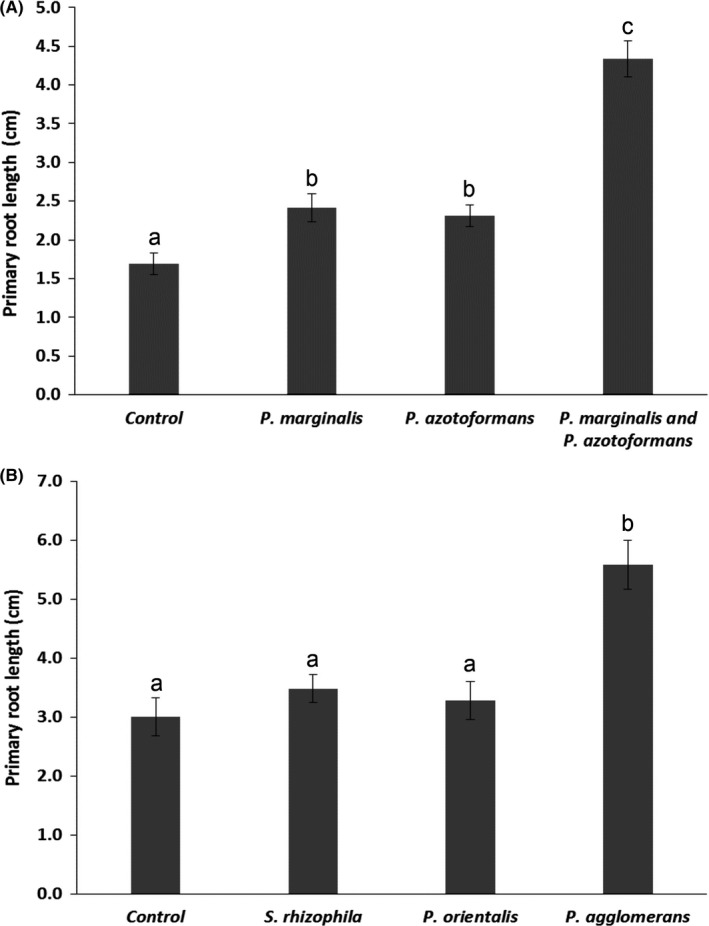
Primary root length (mean ± SE) of all wild cabbage (*B. oleracea*) population seedlings when exposed for five days to bacterial volatiles emitted by (A) *P. marginalis*, *P. azotoformans,* and the combination of both compared with the control (*B. oleracea* without exposure to bacterial volatiles). (B) when exposed to bacterial volatiles emitted by monocultures of *S. rhizophila, P. orientalis,* and *P. agglomerans* or control (no bacterial volatile exposure) for five days. Different letters above bars are based on Tukey HSD multiple comparison tests in general linear model (*n* = 15) and indicate significant differences between the treatments and the control

**Figure 5:**
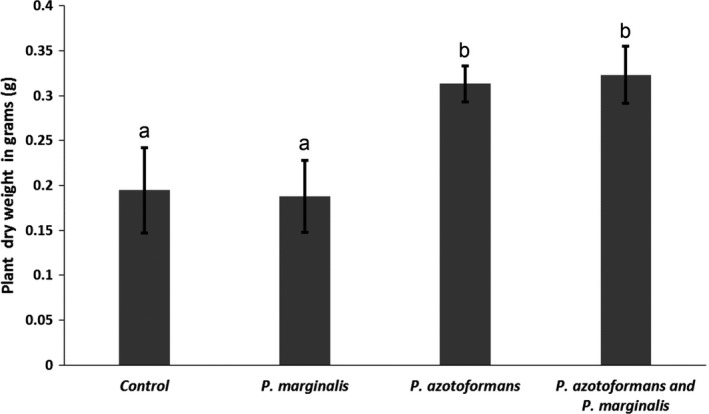
Mean (±SE) dry weight of all wild cabbage plants (*B. oleracea*) when exposed for four weeks to bacterial volatiles emitted by *P. marginalis*, *P. azotoformans,* and the combination of both compared with the control (*B. oleracea* without exposure to bacterial volatiles). Different letters above bars are based on ONE‐WAY ANOVA post hoc Tukey HSD (*p* < .05) and indicate significant differences between the treatments and the control

**Figure A4:**
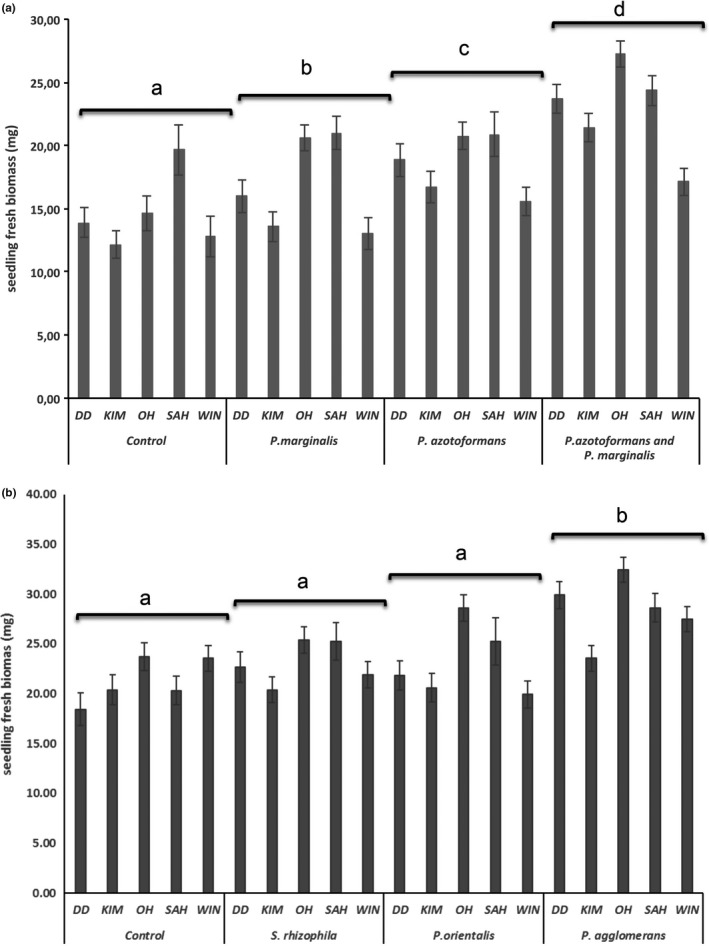
Mean (±SE) seedling fresh biomass (mg) per wild cabbage population (*B. oleracea*) on the 7th day following continuous exposure to bacterial volatiles. (a) *P. marginalis*, *P. azotoformans* and the combination of both compared to the control (*B. oleracea* without exposure to bacterial volatiles); (b) when exposed to bacterial volatiles emitted by monocultures of *S. rhizophila*, *P. orientalis* and *P. agglomerans* or control (no bacterial volatile exposure). Significant differences are indicated by different letters above bars based on Tukey HSD multiple comparison tests in General Linear Model and the interaction with plant populations (*n* = 3), (*p* < .05). Abbreviations for the plant populations: Durdle Door (DD), Kimmeridge (KIM), Old Harry (OH), St. Aldhelms Head (SAH) and Winspit (WIN) [Correction added on 4 December first online publication: Figure A4 has been moved from Supporting Information to the Appendix section]

Volatile exposure treatments significantly affected seed germination (*Chi‐Square* = 38.94; *df* = 3; *p* < .001; Figure 3). Exposure to volatiles of all bacterial monocultures promoted seed germination of all plant populations but this was only significant for the seeds exposed to volatiles of the monocultures of *P. agglomerans* and *P. azotoformans,* as well as to the mixture of *P. marginalis and P. azotoformans* (Figure 3). Seed germination was faster and more seeds were germinated when exposed to volatiles of *P. agglomerans* monocultures in comparison to seed germination in the controls (Figure 3b). When comparing exposure to volatiles produced by a single bacterium species, only volatiles emitted by *P. agglomerans* also strongly promoted primary root length (Figure 4a, b) and seedling fresh biomass (Figures 3a, b, 4a, b) compared with the root length and seedling biomass of the controls and of seeds exposed to volatiles emitted by the other monocultures (root length: *F *= 19.95, *df *= 3; *p *< .001; biomass: general linear model, *df* = 3; *F *= 15.03; *p *< .001). Moreover, bacterial volatiles emitted by the mixture of *P. marginalis* and *P. azotoformans* stronger stimulated seed germination than the bacterial volatiles emitted by the monocultures (Figure 3a) (binary logistic regression, *Chi‐Square* = 290.67; *df* = 3; *p* < .001). Compared with the control treatment, the volatiles emitted by the mixture of *P. marginalis* and *P. azotoformans* also significantly promoted primary root length by a factor of almost three (general linear model, *df* = 3; *F* = 51.22; *p* < .001) (Figure 4a), boosted seedling fresh biomass (Figures 3a, b, 4a, b) (general linear model, *df* = 3; *F* = 35.78; *p* < .001) and plant biomass. Remarkably, there was considerable variability in fresh biomass among the different plant populations of *B. oleracea* exposed to the same volatiles (Figure A4a, b). Dry mass of plants exposed for four weeks to volatiles emitted by *P. azotoformans* and its mixture with *P. marginalis* was significantly higher (0.139 g, *p* = .004) compared with the biomass of the control (0.098 g) when the plants were incubated for four weeks with volatile emitting bacteria (Figure 5). Bacterial volatiles emitted by monocultures of *P. azotoformans* D1 also significantly promoted plant growth of *B. oleracea* (0.148 g, *p* = .029) (Figure 5). No significant growth promotion (0.09 g *p* = .998) was observed for plants after extended exposure to volatiles from monocultures of *P. marginalis* (Figure 5).

### 4.3 Effects of bacterial volatiles on the growth of two plant pathogenic model fungi

**Figure 6:**
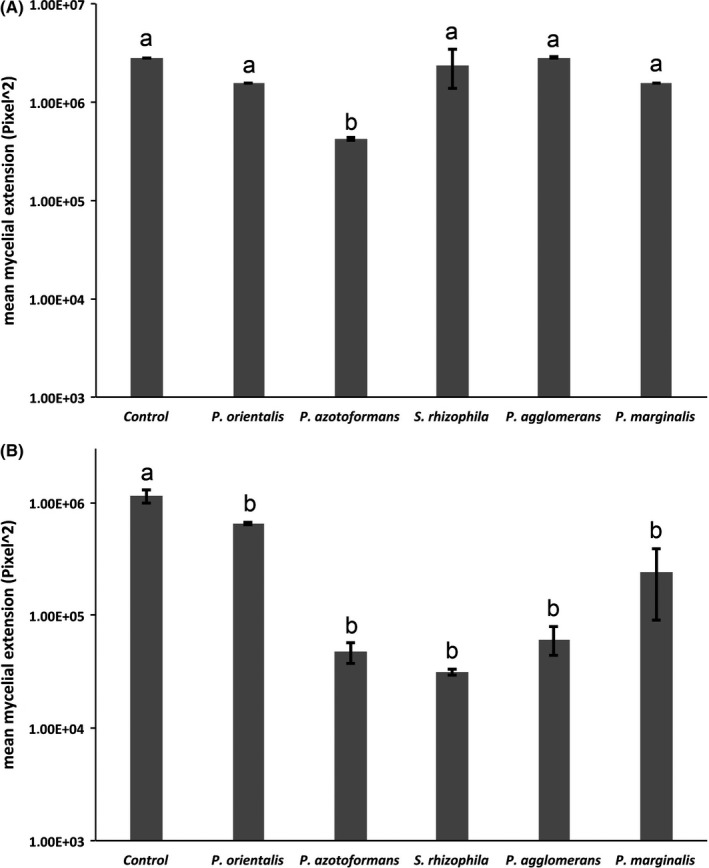
Result of the fungal growth inhibition assay performed with six volatile emitting endophytic bacteria isolated from *B. oleracea* seeds. Bars heights represent the median fungal mycelial extensions; error bars show standard deviation of the mean. (A) Mycelial extension of *R. solani*. (B) Mycelial extension of *F. culmorum*. Significant differences between the control and the treatments are indicated by different letters above bars, based on ONE‐WAY ANOVA post hoc Tukey HSD (*p* < .05)

Volatiles produced by *Pseudomonas azotoformans* D1 were strongly inhibiting (*p = .015*) the growth of the plant pathogenic fungus *Rhizoctonia solani* in comparison to the control. Volatiles emitted by the other four tested bacteria were not able to inhibit the growth of the plant pathogenic fungus *Rhizoctonia solani* significantly (*Pseudomonas marginalis* B1 (*p = .320*) *Pseudomonas orientalis* E8*,* (*p = .333), Stenotrophomonas rhizophila* D5 (*p = .977*), *and Pantoea agglomerans* E44 (*p = 1.000*) (Figure 6a)*.* Interestingly*,* volatiles produced by all five tested endophytic bacteria *(Pseudomonas marginalis* B1 (*p < .001*)*, Pseudomonas orientalis* E8 (*p = .016*)*, Pseudomonas azotoformans* D1 (*p < .001*)*, Stenotrophomonas rhizophila* D5 (*p < .001*), and *Pantoea agglomerans* E44 (*p < .001*) were able to strongly inhibit the growth of the plant pathogenic fungus *Fusarium culmorum* (Figure 6b).

### 4.4 Effects of volatiles emitted by monocultures and mixtures of P. marginalis and P.azotoformans on plant herbivory resistance and larval performance and survival

**Figure A5:**
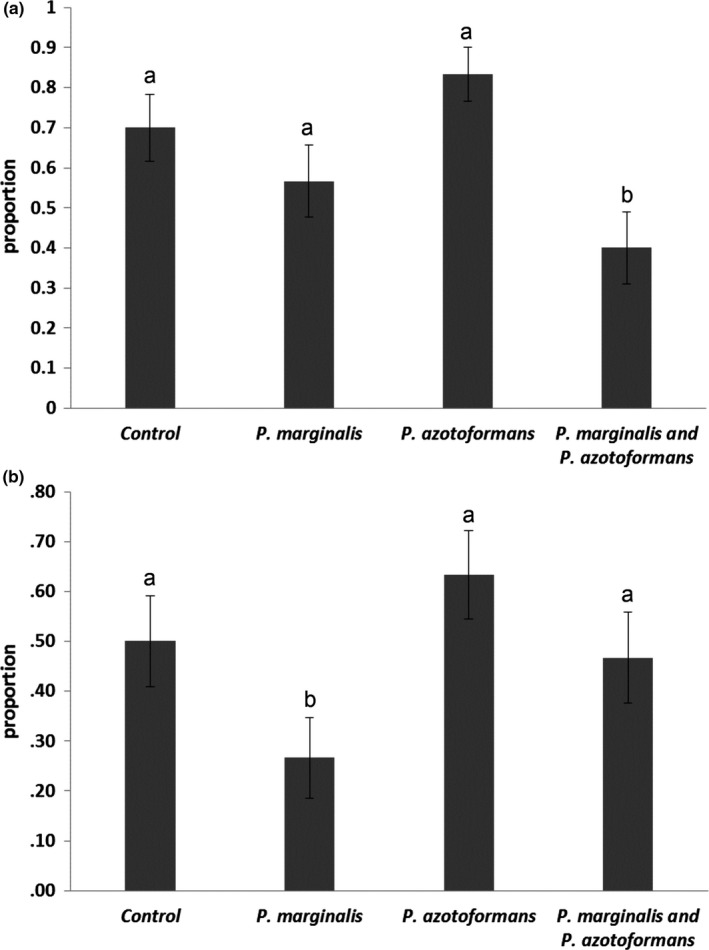
Mean (±SE) larval survival of M. brassicae (proportional) on the (a) 3rd day fed on volatiles‐exposed plants following continuous bacterial volatiles exposure of *P. marginalis* (monoculture), *P. azotoformans* (monoculture) and *P. marginalis* and *P. azotoformans* (mixture) for 29 days and the control (no bacterial volatile exposure) (*n* = 3), Binary Logistic Regression, Chi‐Square = 12.11; *df* = 3; *p* = .007; (b) mean (±*SE*) larval survival of *M. brassicae* (proportional) on the 7th day fed on volatiles‐exposed plants following continuous bacterial volatiles exposure of *P. marginalis* (monoculture), *P. azotoformans* (monoculture) and *P. marginalis* and *P. azotoformans* (mixture) for 29 days or control (no bacterial volatile exposure) (n = 3), Binary Logistic Regression, Chi‐Square = 787.81; *df* = 3; *p* = .049 [Correction added on 4 December 2019 after first online publication: Figure A5 has been moved from Supporting Information to the Appendix section]

Plants from the Winspit population exposed to bacterial volatiles did not significantly affect larval biomass at every time point. The exposure to bacterial volatiles significantly affected larval survival on day 3 (Figure A5a) (*Chi‐Square* = 12.11; *df* = 3; *p* = .007) and on day 7 (Figure A5b) (*Chi‐Square* = 787.81; *df* = 3; *p* = .049). Bacterial volatiles emitted by the monoculture *P. marginalis* B1 and the mixture of *P. marginalis* B1 and *P. azotoformans* D1 but not by the monoculture of *P. azotoformans* D1 reduced survival of the caterpillars.

### 4.5 Detected headspace volatile compounds and effect of interspecific interactions on bacterial volatile blend composition

**Figure 7:**
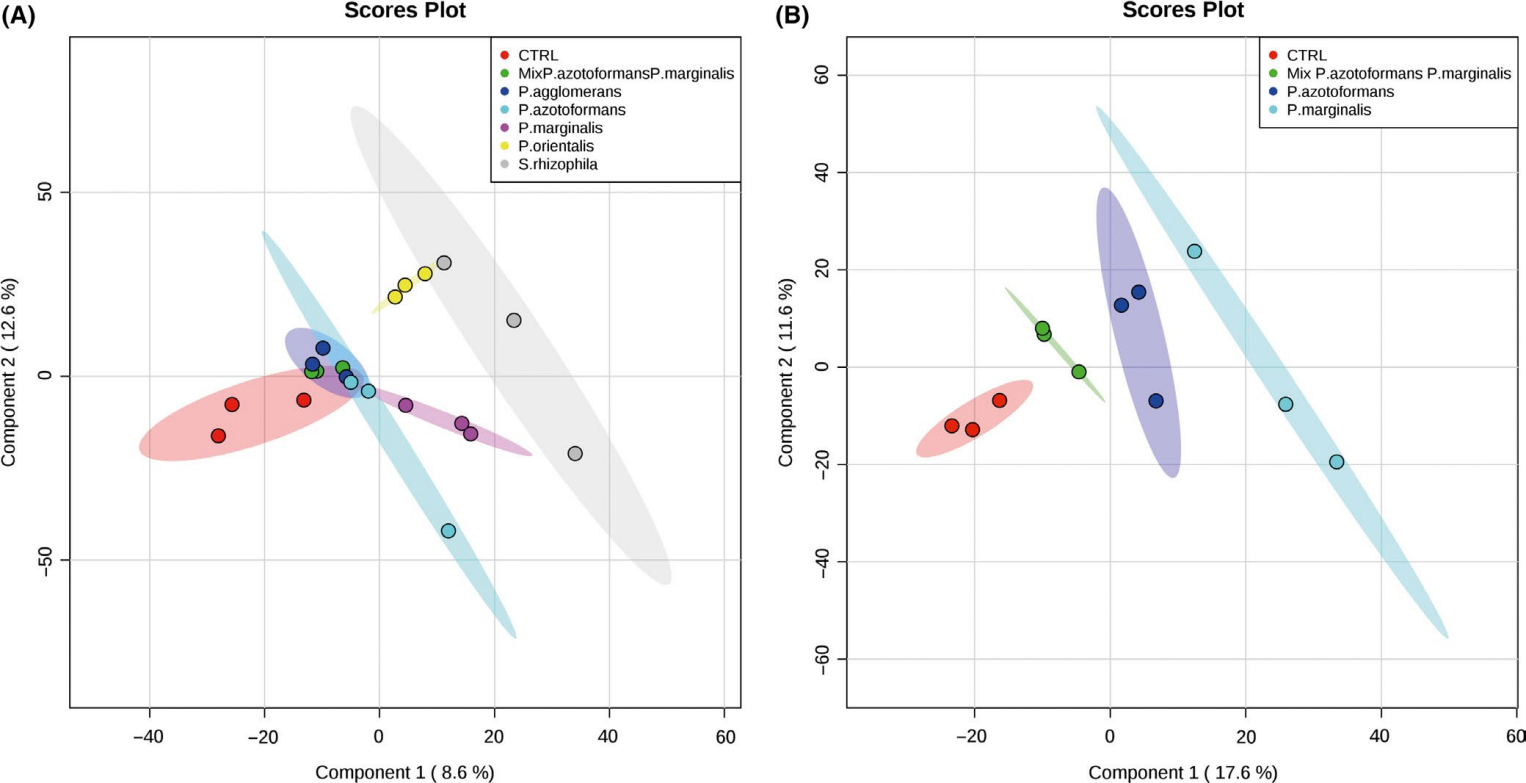
PLSDA 2D‐plot of volatile organic compounds emitted by mono‐ and mixed‐cultures of endophytic bacteria. (A) GC‐MS data obtained from monocultures of *P. agglomerans, P. marginalis*, *P. azotoformans*, *P. orientalis* and *S. rhizophila* and the control (no bacterial volatile exposure) and the mixed culture of *P. azotoformans* and *P. marginalis* (mixture). (B) GC‐MS data of volatiles emitted by the isolates used in herbivory resistance experiments and plant‐growth promoting experiment, monoculture of *P. azotoformans* and *P. marginalis* and the mixture of both

GC/MS‐Q‐TOF analysis revealed a total number of 9 volatile organic compounds that were not detected in the noninoculated controls (Table 2). The 9 detected compounds belonged to different chemical classes including acids, alcohols, alkenes, terpenes, and sulfides. Each bacterium emitted its specific blend of compounds and the emitted individual volatiles compounds differed between each bacterial inoculum (Table 2, Figure 7a). The PLSDA analysis could clearly separate the blends**.** Clear separations between controls, monocultures, and the combination of *P. marginalis* with *P. azotoformans* were obtained in PLSDA score plots (Figure 7a, b). The volatile composition of the blend emitted by the bacterial mixture resembled that of the blends emitted by the monocultures of these bacteria. Three compounds, cyclohexane, dimethyl disulfide, dimethyl trisulfide were emitted by all bacterial inocula. We could tentatively identify 7 compounds emitted by monocultures of *P. agglomerans E44*, 6 for *P. marginalis B1*, 7 for *P. azotoformans D1*, 6 for *S. rhizophila D5*, and 4 for *P. orientalis E8*. For the combinations of *P. marginalis* with *P. azotoformans,* we obtained a total number of 7 volatile organic compounds. The most prominent detected headspace volatile organic compounds were the two sulfur‐containing compounds dimethyl disulfide (C_2_H_6_S_2_) and dimethyl trisulfide (C_2_H_6_S_3_) that were produced by all tested bacteria (Table 2). Interestingly, 1‐undecene and the unknown compound produced by the monoculture of *P. marginalis* were not detected in the blend produced by the bacterial mixture (Table 2).

**Table 2:** Tentatively identified volatile organic compounds (VOCs) produced by endophytic bacteria isolated from seeds of B. oleracea

						**Detected in treatment**					
#	Compound name	RT*	ELRI**	p‐value***	Chemical family	PA	PSM	PSA	SR	PO	PSM + PSA
1	Cyclohexane	3.36	718	1.70E−15	Alkenes	X	X	X	X	X	X
2	1‐pentanol	4.60	753	2.60E−11	Alcohols	—	—	X	X	—	X
3	dimethyl disulfide	4.83	759	3.00E−05	Sulfides	X	X	X	X	X	X
4	alpha‐pinene	11.20	930	2.03E−19	Terpenes	X	—	X	X		X
5	dimethyl trisulfide	12.60	963	1.44E−06	Sulfides	X	X	X	X	X	X
6	1‐undecene	18.10	1,092	1.95E−04	Alkenes	X	X	—	—	X	—
7	unknown terpene like compound	29.83	1,409	2.12E−04	‐	X	X	X	—	—	X
8	unknown compound	35.80	1,600	1.61E−15	‐	X	X	—	X	—	—
9	hexadecanoic acid	40.70	1,948	2.12E−04	Acids	—	—	X	—	—	X
Number of compounds (*n*)						7	6	7	6	4	7

Abbreviations: PA, *Pantoea agglomeran*; PSM, *Pseudomonas marginalis*; PSA, *Pseudomonas azotoformans;* SR, *Stenotrophomas rhizophila*; PSO, *Pseudomonas orientalis*; PSM + PSA, *Pseudomonas marginalis* + *Pseudomonas azotoformans.*

RT*, retention time, the RT value stated is the average retention time of three replicates.

ELRI**, experimental linear retention index value, the RI value stated is the calculated average of three replicates.

p‐value***, statistical significance (peak area and peak intensity).

## 5 DISCUSSION

Seeds and plant seedlings are clearly a crucial stage of a plant's development: failure to germinate is lethal. However, thus far, little is known about seed‐associated microorganisms and their impacts on plant growth and development (Nelson, 2018). Furthermore, there is not much knowledge about the metabolites produced by the microorganisms that reside inside seeds and their effect on plant development, growth, and health. Despite increasing awareness of the importance of the plant holobiont to plant evolution and ecology, the importance of the seed microbiome has generally been neglected (Berg & Raaijmakers, 2018; Hacquard, 2016; Rosenberg & Zilber‐Rosenberg, 2016). This is one of the few studies investigating the beneficial effects of seed‐associated bacteria and the metabolites produced by these bacteria on plant development, growth, and health. The number of colony‐forming units (CFU/ g of seed material) obtained from five different plant populations of *B. oleracea* (10^2 – 10^3 CFU/g of seed material) is in line with results of other studies investigating the abundance of endophytic bacteria in plant tissues (Compant, Mitter, Colli‐Mull, Gangl, & Sessitsch, 2011; Ferreira et al., 2008; Graner, Persson, Meijer, & Alstrom, 2003; Rosenblueth et al., 2012; Truyens et al., 2015). Many of the bacteria isolated from the seeds of *B. oleracea* belonged to the genera *Chyrseobacterium*, *Stenotrophomonas*, *Sphingomonas*, *Pseudomonas* and *Pantoea*, which are known bacterial endophytes of many plant species (Graner et al., 2003; Nelson, 2004; Truyens et al., 2015). However, our study focused on culturable bacteria and, therefore, only a subset of the total seed‐associated microbiome was assessed. Further metagenome‐based studies need to be performed to detect the other nonculturable microorganisms associated with plant seeds.

Bacterial volatiles emitted by each of the monocultures stimulated seed germination in comparison to the control. The observation that bacterial volatiles are able to promote plant growth is already known (Bailly & Weisskopf, 2012; Blom et al., 2011; Kanchiswamy et al., 2015; Xie, Zhang, & Pare, 2009); however, the observation that bacterial volatiles promote seed germination has not been reported before. Interestingly, only bacterial volatiles from the monoculture *P. agglomerans* and the mixture of *P. marginalis* and *P. agglomerans* strongly promoted primary root length seedling fresh biomass. Bacteria, such as *P. agglomerans,* have previously been shown to promote plant growth (Hernández‐León et al., 2015; Quecine et al., 2012; Santoyo, Orozco‐Mosqueda, & Govindappa, 2012; Vespermann, Kai, & Piechulla, 2007). However, the effects of volatiles emitted by this bacterial species on plant growth and development have not been reported before. The mixture of *P. marginalis* and *P. azotoformans* strongly enhanced plant dry biomass compared with the monocultures, suggesting that there was a synergistic effect of these two bacteria on plant growth. The bacteria involved in plant‐growth promotion belong to *Pseudomonas* species, bacteria of these species are well‐known for their plant‐growth promoting effects (Park et al., 2015; Raza, Yousaf, & Rajer, 2016; Santoyo et al., 2012). The mechanism underling growth‐promoting effects of bacterial volatiles are largely unknown. It has been proposed that bacterial volatiles may modulate phytohormonal networks in the host plants, such as those involving ethylene (Ryu et al., 2003), cytokinin (Ortiz‐Castro, Valencia‐Cantero, & Lopez‐Bucio, 2008), ABA (Zhang et al., 2008) or auxin (Bailly et al., 2014). However, the target tissues of bacterial volatiles and how these are recognized and activate plant signaling are still being investigated (Bailly & Weisskopf, 2012; Sharifi & Ryu, 2018).

In general, whereas all five of the cabbage populations performed better when exposed to bacterial volatiles, three of the populations stood out in this regard. The Kimmeridge (plant population B) and the Old Harry (plant population C) population showed better seed germination and seedlings produced longer primary roots (data not shown). Furthermore, The St. Aldhelms Head (plant population D) and the Old Harry population yielded higher seedling fresh biomass compared with the three other populations. Overall, the Old Harry population showed the best plant performance regardless of the bacterial volatile blend it had been exposed to. These results suggest that seeds of the various cabbage populations differ in their responsiveness to growth promotion by bacterial volatiles. Interestingly, we could only isolate one bacterial species that could be cultured from the Old Harry plant population. However, this might be due to the applied culture‐dependent approach and, most probably, only subsets of the total seed microbiome of each plant population has been assessed.

Furthermore, this study investigated how the exposure to volatiles emitted by the endophytic bacteria influenced the resistance of wild *B. oleracea* plants to *M. brassicae* larvae. The highest mortality was found when *M. brassicae* larvae were exposed to volatiles emitted by the bacterial mixture of *P. marginalis* and *P. azotoformans* after three days and by the monocultures of *P. marginalis* after seven days. These results suggest that plants exposed to bacterial volatiles has only marginal and transient effects on the larval performance. It is also possible that the larvae are less affected by increased plant resistance as their development advances (Jeschke et al., 2017). Previous work demonstrated that exposure of *Arabidopsis* to volatiles emitted by *Bacillus amyloliquefaciens* GB03 transcriptionally induced sulfate assimilation, and this resulted in increased total shoot glucosinolates and reduced larval performance of *Spodoptera exigua* (Aziz et al., 2016). In the study (Aziz et al., 2016), larval performance was also determined when caterpillars had been feeding on the plants for 7 and 9 days, respectively, and did not cover complete immature development (larvae may compensate for initial reduced feeding later in their development).

The five British wild cabbage populations studied here grow along a linear transect along the often rugged chalky coastline of Dorset and geographic formations known as the “Purbeck Hills”. These populations are discrete and apparently have been stable for many decades and perhaps centuries (Wichmann et al., 2008). Previous studies have shown that concentrations and types of secondary metabolites in them (glucosinolates) differ markedly among the different populations, even those growing within a few km of each other (Gols et al., 2009, 2008; Moyes et al., 2000). This suggests that there may be little gene flow between them (Wichmann et al., 2008). The five populations also exhibit varying degrees of exposure to prevailing winds from the south to west, which are often persistent and reach gale force in the more exposed locations (e.g. St. Aldhelms Head and Kimmeridge). Moreover, some of the plant populations are not that large: Old Harry, for instance, contains ~ 50–100 plants, many of them at least several years old (Mitchell & Richards, 1979). The vegetation has been classified as maritime grassland and the floral diversity largely depends on the degree of exposure to harsh conditions (Mitchell & Richards, 1979; Wichmann et al., 2008). The species is considered a poor competitor and seedlings are easily shaded out by grasses in spring such as *Festuca rubra* and *Lolium perenne* (Mitchell & Richards, 1979). Therefore, the presence of endophytic bacteria on seeds may play a crucial role in enabling wild cabbage to persist in the face of intense competition with grasses for germination sites.

This is the first report showing how wild cabbage populations respond toward bacterial volatiles coming from their own seed microbiome. Our study clearly shows that seeds endophytes may play an important role in early development of the plant (seed germination and seedling growth). This study indicates the importance to further explore the seed‐associated microbiome and the interactions within the seed microbiome and between the seed microbiome and the host plant. Further studies should combine both metagenomics and culturable approaches in order to comprehensively understand the underlying mechanism of positive impacts of the seed microbiome on plant growth, development, and resistance in wild cabbage plants.

## CONFLICT OF INTERESTS

None declared.

## AUTHOR CONTRIBUTIONS

OT and RP designed the experiments. OT and RP performed the lab experiments. RP and OT performed the data analysis and prepared the figures and tables. RG provided insects larvae. RP, OT, RG, JH and PG wrote the manuscript. OT, RP, RG, JH and PG contributed to the revision of the manuscript.

## ETHICS STATEMENT

None required.

## Figures and Tables

**Figure A3:**
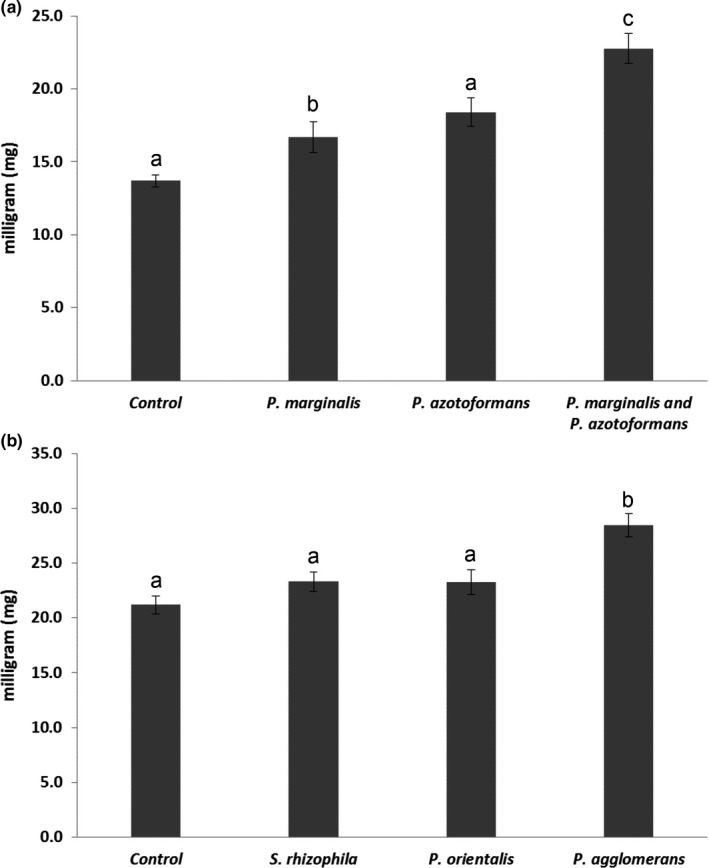
Mean (±SE) seedling fresh biomass (mg) of wild cabbage plants (*B. oleracea*) of all wild cabbage population when exposed to bacterial volatiles emitted for seven days. (a) *P. marginalis*, *P. azotoformans* and the combination of both compared to the control (*B. oleracea* without exposure to bacterial volatiles); (b) when exposed to bacterial volatiles emitted by monocultures of *S. rhizophila*, *P. orientalis* and by different letters above bars based on Tukey HSD multiple comparison tests in General Linear Model (*n* = 15), (*p* < .05) [Correction added on 4 December 2019 after first online publication: Figure A3 has been moved from Supporting Information to the Appendix section]

## Data Availability

All 16S rRNA gene sequences obtained in this work were submitted to the NCBI Genbank database (https://www.ncbi.nlm.nih.gov/genbank/) with accession numbers MN079062 – MN079072.
